# Changes in physical demands between game quarters of U18 elite official basketball games

**DOI:** 10.1371/journal.pone.0221818

**Published:** 2019-09-03

**Authors:** Jairo Vázquez-Guerrero, Bruno Fernández-Valdés, Ben Jones, Gerard Moras, Xavi Reche, Jaime Sampaio

**Affiliations:** 1 Sport Performance Department, FC Barcelona Sports, Barcelona, Spain; 2 National Institute of Physical Education of Catalonia (INEFC), Barcelona, Spain; 3 Unió Esportiva Santboiana, DH Rugby, Sant Boi de Llobregat, Barcelona, Spain; 4 Institute for Sport, Physical Activity and Leisure, Leeds Beckett University, Leeds, United Kingdom; 5 Yorkshire Carnegie Rugby Club, Headingley Carnegie Stadium, Leeds, United Kingdom; 6 Leeds Rhinos Rugby Club, Headingley Carnegie Stadium, Leeds, United Kingdom; 7 England Performance Unit, The Rugby Football League, Leeds, United Kingdom; 8 School of Science and Technology, University of New England, Armidale, NSW, Australia; 9 Division of Exercise Science and Sports Medicine, Department of Human Biology, Faculty of Health Sciences, the University of Cape Town and the Sports Science Institute of South Africa, Cape Town, South Africa; 10 Research Center in Sport Sciences, Health and Human Development (CIDESD), CreativeLab Research Community, University of Trás-os-Montes e Alto Douro, Vila Real, Portugal; Universidade Federal de Juiz de Fora, BRAZIL

## Abstract

**Purpose:**

The aim of this study was to describe the physical demands during U18 elite basketball games according to the game quarter and to identify a smaller subset of variables and threshold scores that distinguish players’ physical performance in each quarter.

**Methods:**

Data was collected from ninety-four players who participated in the study (age: 17.4 ± 0.74 years; height: 199.0 ± 0.1 cm; body mass: 87.1 ± 13.1 kg) competing in the Euroleague Basketball Next Generation Tournament. Players’ movements during the games were measured using a portable local positioning system (LPS) (WIMU PRO®, Realtrack Systems SL, Almería, Spain) and included relative distance (total distance / playing duration), relative distance in established speed zones, high-intensity running (18.1–24.0 km·h-1) and sprinting (> 24.1 km·h^-1^). player load, peak speed (km·h^-1^) and peak acceleration (m·s^-2^) number of total accelerations and total decelerations, high intensity accelerations (> 2 m·s^-2^) and decelerations (< -2 m·s^-2^).

**Results:**

There was an overall decrease in distance covered, player load, number of high intensity accelerations and decelerations between the first and last quarter of the games in all playing positions. A classification tree analysis showed that the first quarter had much influence of distance covered (above 69.0 meters), distance covered <6.0 km·h^-1^ and accelerations (> 2 m·s^-2^), whereas the fourth quarter performance had much influence of distance covered (below 69.0) and distance covered 12.1–18.0 km·h^-1^.

**Conclusions:**

A significant reduction in physical demands occurs during basketball, especially between first and last quarter for players in all playing positions during basketball games of under 18 elite players.

## Introduction

Basketball is an intermittent, dynamic and complex team sport where multidirectional bursting movements such as accelerations, impacts, jumps, sprints are combined with technical and tactical offensive and defensive actions. As such, during the game there are biomechanical, neuromuscular and physiological demands [1] comprising around 115 actions that require maximum intensity [2].

Available research has developed time-motion methods based on computer vision tracking technology, that can be quite time consuming, and whose results have been controversial. For example, in rugby competition the total time spent in sprinting, striding, jogging, walking, static exertion and being stationary showed moderate to poor reliability (5.8–11.1% TEM—typical error of measurement [3]) In basketball, the total time spent covering distances at high (from 2 to 20%), moderate (from 17 to 68%) and low intensity (from 30 to 72%) can also change considerably [4–6]. In the last years, Global Positioning Systems (GPS) have allowed to measure physical demands in training and competition for outdoor team sports like rugby and soccer [7], enabling coaches and sports scientists to obtain more detailed information.

As an indoor team sport, basketball physical demands during games are scarcely known, probably due to the lack of monitoring methodologies to achieve valid and reliable data. In the last years, studies are using inertial micro sensors, such as accelerometers, to measure the activity in non-elite competitive basketball games [8]. More recently, research has described the activity using GPS combined with accelerometer sensors, but using non-elite basketball competitive games [9].

These micro-technology advances have allowed to use local positioning systems (LPS) to track players indoor, providing better levels of validity and reliability than the standard GPS systems [10]. In basketball, research using LPS has analyzed the physical demands of defensive pressure on half-court and full-court and how it affects collective behaviors during simulated games [11]. Another study has reported that physical demands can be modulated modifying the rules playing 5 on 5 scrimmage drills, suggesting that this factor should be taken into account when programing training drills and training loads [12]. Although these studies present novel information, they are constrained by the usage of non-professional players during simulated games and practice conditions. Nevertheless, they also suggest that acceleration-derived variables can complement the more conservative time motion analyses.

Acceleration precedes high speed running and requires high rates of force development, but it is a distinct phenomenon [13] which requires higher neural activity of the working muscles than constant speed sprinting [14] as described by Akenhead et al. (2013) [15]. This is a key activity to obtain advantages during team sports [16], therefore, the quantification of high intensity accelerations and decelerations has gained attention [17,18]. Previous research, has identified the total number of accelerations and decelerations during basketball to determine different zones of intensity during professional basketball games [18]. Furthermore, accelerations and decelerations may be produced in different axes that might correspond to different skills, such as sprinting and changing direction. Also, a higher number of maximal decelerations can largely increase the mechanical stress imposed on the players and may be associated with a greater eccentric muscular work, that should be included in strength and conditioning programs and injury prevention programs [18].

Research in basketball has identified the effect of the starting score-line on the game quarter final score [19], suggesting that a greater difference in the accumulated score at the beginning of each quarter would correspond with a higher number of recovered points by the losing teams. These results also suggest that inter-quarter variability in activity demands might be very high, due to these different baseline conditions. For example, a team that enters the third quarter in a game winning by eight points will likely have a less physically demanding quarter than if it enters the quarter losing by eight points. Therefore, because these game scenarios are unpredictable, coaching staffs will benefit from knowing the variables that best describe the physical demands in each quarter and, also, the threshold scores of these variables. Unfortunately, research is very scarce on providing these details that can really help coaching staffs and players.

Therefore, the aim of this study was to describe the physical demands during U18 elite basketball games according to the game quarter and to identify a smaller subset of variables and threshold scores that distinguish players’ physical performance in each quarter. The knowledge of these requirements can help coaches and strength and conditioning trainers to design more adequate training sessions and conditioning programs, specifically by understanding better the dynamics from the motion demands in each game quarter.

## Materials and methods

### Subjects

The subjects were part of eight teams from six countries professional men’s basketball teams, competing in the Euroleague Basketball Next Generation Tournament. Ninety-four male subjects participated in the study (age: 17.4 ± 0.74 years; height: 199.0 ± 0.1 cm; body mass: 87.1 ± 13.1 kg) from different playing positions, Guards (n = 35), Forwards (n = 42) and Centers (n = 17). The Tournament was played from 18 to 21 May 2017. All of the players and coaches were informed about the research protocol, requirements, benefits, and risks, and their written consent was obtained before the study began. Basketball team positional roles were classified into three playing specific-positions: guards, forwards and centers [20]. The study protocol followed the guidelines and was approved by the local Ethics Committee (UID/DTP/04045/2013), Research Center in Sports Sciences, Health Sciences and Human Development, CIDESD, Geron Research CommunityUniversity of Trás-os-Montes and Alto DouroVila Real, Portugal, and conformed to the recommendations of the Declaration of Helsinki.

### Design and methodology

A non-experimental descriptive design was used to examine the differences between physical demands for playing positions during competitive matches. The players’ motion activity was assessed using WIMU PRO Local Positioning System (Realtrack Systems, Almeria, Spain). A total of thirteen games were monitored over the 4-day tournament. All games were played in the same court based on International Basketball association (FIBA) rules and started with an official 15-minute warm-up, based on ball dribbling, shooting, specific mobility and dynamic stretching exercises. The motion demands were quantified only when the players were competing on court, i.e., excluding the warm up, time on bench and rest time between quarters. The players were able to replace water loss by drinking *ad libitum* during recovery periods.

Players’ data were included for analysis provided they met the following criteria: (i) they did not suffer injury during the game and (ii) they played in the same position throughout the game, and (iii) they played at least 5 minutes in each game. Playing positions were first determined by the information available on the Euroleague website, and further refined by a qualified basketball coach. With these inclusion criteria, 266 observations were included in the study: Guards (*n* = 104), Forwards (*n* = 119), and Centers (*n* = 43).

Players’ movements were measured using a portable LPS (WIMU PRO, Realtrack Systems SL, Almería, Spain) during games. These devices were fitted to the upper back of each player using an adjustable harness (Rasán, Valencia, Spain). The WIMU PRO units integrate different sensors registering at different sample frequencies. Sampling frequency for 3-axis accelerometer, gyroscope and magnetometer was 100 Hz and 120 kPa for the barometer. The system has six ultra-wideband antennas, 4 placed 3 meters outside the corners of the court and 2 placed 3 meters outside half-court, the sampling frequency for positioning data were 20 Hz. The system operates using triangulations between the antennas and the units, the six antennas send a signal to the units every 50 ms. Then, the device calculates the time required to receive the signal and derives the unit position (coordinates X and Y), using one of the antennas as a reference. Data were analyzed using the system specific software (SPRO Software, Realtrack Systems SL, Almería, Spain).

The following variables were measured and reported: A) relative distance (total distance/playing duration); and B) relative distance in established speed zones: stationary/walking (<6.0 km·h^-1^), jogging (6.0–12.0 km·h^-1^), running (12.1–18.0 km·h^-1^), high-intensity running (18.1–24.0 km·h^-1^) and sprinting (>24.1 km·h^-1^). These speed and movement zones are similar to those used in other basketball studies [21]; C) Player load, vector magnitude expressed as the square root of the sum of the squared instantaneous rates of change in acceleration in each of the three planes divided by 100 [22]; D) Peak speed (km·h^-1^) and peak acceleration (m·s^2^), as the highest value obtained during the situation. E) number of total accelerations and total decelerations, and F) the number of jumps that exceed 3 G’s forces, measured with the inertial accelerometer in the Z and X, Y axis respectively, high intensity accelerations (> 2 m·s^-2^) and decelerations (< -2 m·s^-2^).

WIMU PRO system showed better accuracy (bias: 0.57–5.85%), test–retest reliability (%TEM: 1.19), and inter-unit reliability (bias: 0.18) in determining distance covered compared to GPS technology (bias: 0.69–6.05%; %TEM:1.47; bias: 0.25) overall [23]. Also, it showed better results (bias: 0.09; ICC: 0.979; bias: 0.01) for mean velocity measurement than GPS (bias: 0.18; ICC: 0.951; bias: 0.03)[23].

### Statistical analyses

Descriptive statistics (mean ± SD) for the outcome measures were calculated. A classification tree analysis was used to determine game quarter classification according to all the considered performance variables. This technique consists in splitting the sample into different sub-groups (nodes) based on the impact of all predictors (i.e., distance covered, accelerations, player load,…) on the classification of the game quarter (i.e., first to fourth). In addition, this technique will provide visual information about the impact of each independent variable in a hierarchical tree model [24]. The algorithm used was the exhaustive CHAID (Chi-squared automatic Interaction detection), that uses chi-square tests to identify the relationships between independent variables through completing three steps on each node of the root (merging, splitting and stopping), in order to identify predictors that exert the most influence on the dependent variable (game quarter). The algorithm examines all possible splits for each predictor and the merging step increases the search procedure to merge any similar pair until only single pair remains [25]. The following statistical specifications were considered: (i) maximum three depth was 3; (ii) minimum cases in parent and child node were 80 and 40, respectively; (iii) maximum number of iterations were 100; (iv) the minimum change in expected cell frequencies is 0.001. The risk of misclassification was calculated as a measure of the overall reliability of the model [25]. At the end, results from terminal nodes were contrasted with playing positions (guards, forwards and centers), tournament outcome (best four classified teams and worst four classified teams), game type (games played between the best teams, between the worst teams and other games) and team, using qui-squared tests.

Also, a linear mixed-effects model was used to model the main and interactive effects using PASW Statistics 21 (SPSS, Chicago, USA). “quarter” (Q1, Q2, Q3 or Q4) and “position” (Center, Forward or Guard) were treated as the fixed effects, whereas the random effects were “ID Player” and “match-code”. Then Magnitude-based inferences were applied using the estimates from the linear mixed model (representing percentage differences between sections by positions) and were compared against a smallest worthwhile effect threshold equivalent to 0.2 of the between-subject standard deviations using a spreadsheet [26]. Effects were classified as unclear if the percentage likelihood that the true effect was positive and negative were both >5%. Otherwise, the effect was deemed clear, and was qualified with a probabilistic term for increase or decrease using the following scale: <0.5%, most unlikely; 0.5–4.9%, very unlikely; 5–24.9%, unlikely; 25–74.9%, possible; 75–94.9%, likely; 95–99.5%, very likely; >99.5%, almost certainly [27]. Also, the comparisons were assessed via standardized mean differences (Cohen´s d) and respective 90% confidence intervals. Thresholds for effect sizes statistics were <0.20, trivial; 0.20–0.59, small; 0.6–1.19, moderate; 1.20–1.99, large; and >2.0, very large [27].

## Results

The activity demands for guards, forwards and centres in each game quarter are shown in Table 1.

**Table 1:** Means (±SD) in selected physical demands for positions.

Physical demands	Quarters	Center	Forward	Guard
distance covered	Q1	73.45 ± 12.97	78.91 ± 10.09	80.46 ± 7.57
	Q2	69.10 ± 7.97	71.90 ± 9.03	73.91 ± 8.93
	Q3	68.95 ± 9.45	71.98 ± 11.23	76.81 ± 8.46
	Q4	64.24 ± 8.50	69.15 ± 13.87	70.00 ± 9.89
< 6 (km · h^-1^)	Q1	30.12 ± 4.66	29.84 ± 3.41	30.30 ± 3.06
	Q2	29.52 ± 3.58	28.95 ± 3.40	28.94 ± 2.86
	Q3	31.44 ± 3.89	30.23 ± 3.96	30.69 ± 3.07
	Q4	29.64 ± 3.90	28.92 ± 2.81	29.27 ± 2.98
6.0–12.0 (km · h^-1^)	Q1	26.26 ± 5.83	28.97 ± 4.88	30.13 ± 4.82
	Q2	22.48 ± 3.99	25.01 ± 4.65	27.57 ± 4.93
	Q3	23.06 ±4.77	24.88 ± 5.54	28.27 ± 4.40
	Q4	22.00 ±3.98	24.07 ± 6.01	25.55 ± 5.40
12.1–18.0 (km · h^-1^)	Q1	14.42 ± 5.38	16.64 ± 5.22	16.73 ± 4.20
	Q2	14.69 ± 4.91	15.19 ± 4.54	14.74 ± 4.04
	Q3	12.54 ± 3.86	14.60 ± 4.52	14.99 ± 4.34
	Q4	10.80 ± 3.67	13.93 ± 7.58	12.92 ± 4.46
18.1–24.0 (km · h^-1^)	Q1	2.56 ± 1.34	3.24 ± 2.05	3.14 ± 1.71
	Q2	2.38 ± 1.45	2.64 ± 1.74	2.45 ± 1.50
	Q3	1.85 ± 1.56	2.21 ± 1.51	2.72 ± 1.48
	Q4	1.77 ± 1.71	2.14 ± 1.92	2.17 ± 1.60
> 24.1 (km · h^-1^)	Q1	0.08 ± 0.19	0.22 ± 0.34	0.16 ± 0.35
	Q2	0.03 ± 0.12	0.11 ± 0.24	0.22 ± 0.41
	Q3	0.06 ± 0.18	0.06 ± 0.19	0.15 ± 0.29
	Q4	0.03 ± 0.09	0.09 ± 0.24	0.10 ± 0.24
Player load (a.u.)	Q1	1.44 ± 0.29	1.49 ± 0.28	1.50 ± 0.18
	Q2	1.36 ± 0.22	1.33 ± 0.19	1.36 ± 0.22
	Q3	1.32 ± 0.19	1.34 ± 0.26	1.40 ± 0.19
	Q4	1.26 ± 0.26	1.26 ± 0.17	1.27 ± 0.22
Peak speed (km · h^-1^)	Q1	19.16 ± 0.89	19.35 ± 1.05	19.57 ± 0.97
	Q2	18.82 ± 1.09	39.34 ± 1.03	19.56 ± 1.32
	Q3	18.75 ± 1.04	18.92 ± 2.34	19.64 ± 0.88
	Q4	19.07 ± 0.96	19.15 ± 1.03	19.39 ± 1.01
Peak accelerations (m·s^-1^)	Q1	3.21 ± 0.28	3.38 ± 0.32	3.47 ± 0.28
	Q2	3.15 ± 0.29	3.37 ± 0.33	3.46 ± 0.32
	Q3	3.12 ± 0.31	3.29 ± 0.31	3.49 ± 0.24
	Q4	3.07 ± 0.31	3.33 ± 0.31	3.43 ± 0.26
Accelerations > 2 (m·s^-1^)	Q1	1.76 ± 0.69	2.04 ± 0.65	2.20 ± 0.48
	Q2	1.64 ± 0.47	1.83 ± 0.54	1.99 ± 0.61
	Q3	1.44 ± 0.37	1.72 ± 0.50	1.95 ± 0.53
	Q4	1.26 ± 0.55	1.66 ± 0.61	1.72 ± 0.43
Decelerations < -2 (m·s^-2^)	Q1	1.25 ± 0.48	1.70 ± 0.59	2.04 ± 0.48
	Q2	1.20 ± 0.44	1.47 ± 0.51	1.79 ± 0.51
	Q3	1.04 ± 0.34	1.39 ± 0.50	1.82 ± 0.51
	Q4	0.99 ± 0.40	1.28 ± 0.50	1.52 ± 0.44

**Fig 1:**
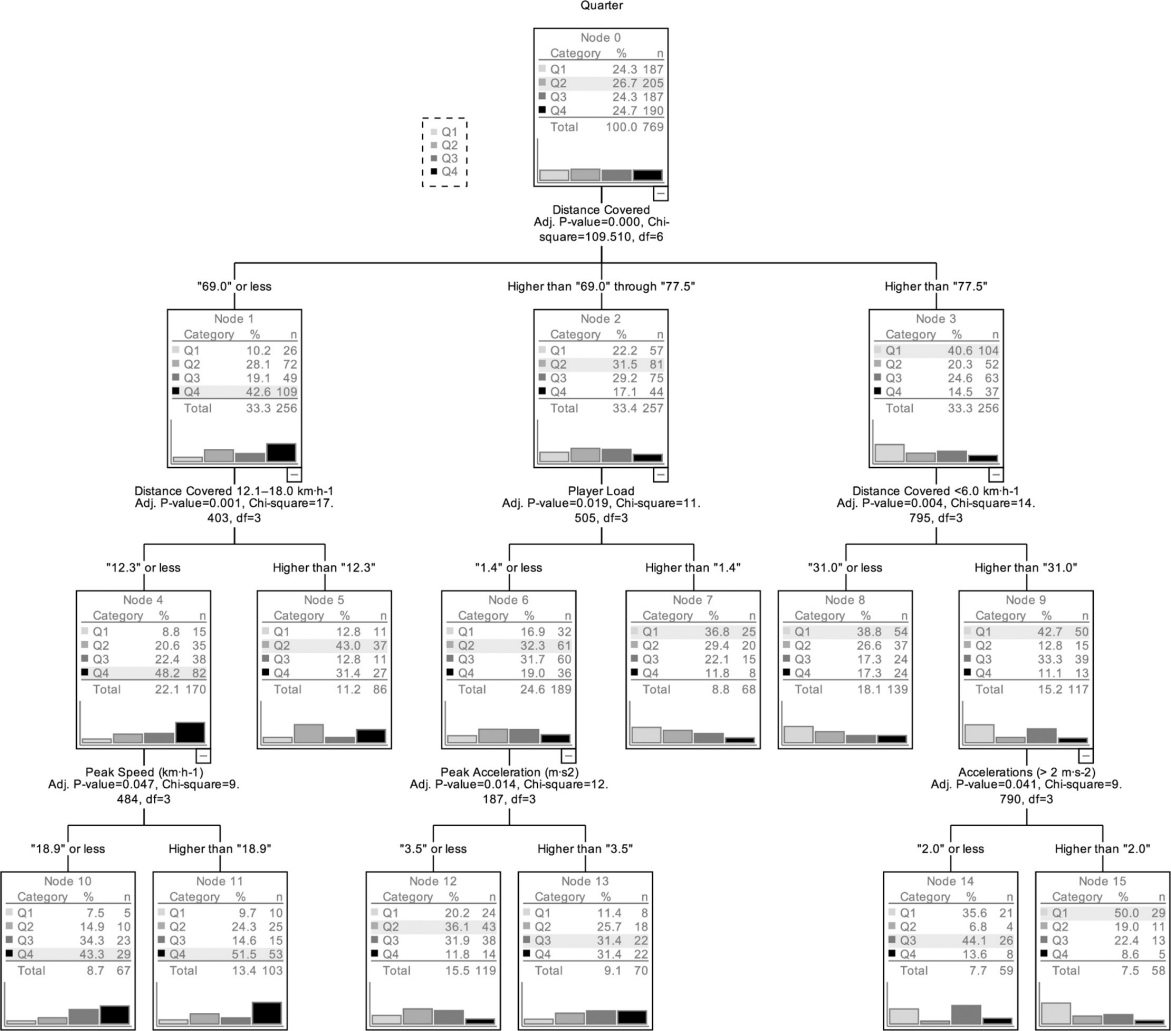
Classification tree from all variables according to the game quarter.

Fig 1 presents the obtained classification tree, that gathers 9 terminal nodes from a total of 16 nodes. The overall reliability of the model was 0.586±0.018, for a total percentage of correct classification of 41.1%. For each game quarter, the correct classifications were 57.8%, 39.0%, 25.7% and 43.2%, respectively. The distance covered was the stronger predictor, game performances with distance covered of 69.0 of less occurred mainly in the fourth quarter, whereas game performances higher than 77.5 occurred mainly in the first quarter. The first quarter had predominance in terminal nodes 7.8 and 15, with much influence of distance covered (above 69.0), distance covered < 6.0 km·h^-1^ and accelerations (> 2 m·s^-2^). The fourth quarter performance was very distinct, the predominance is in terminal nodes 10 and 11, with much influence of distance covered (bellow 69.0) and distance covered 12.1–18.0 km·h^-1^. The second quarter was best described by distance covered between 69.0 and 77.5 and player load less than 1.4.

**Fig 2:**
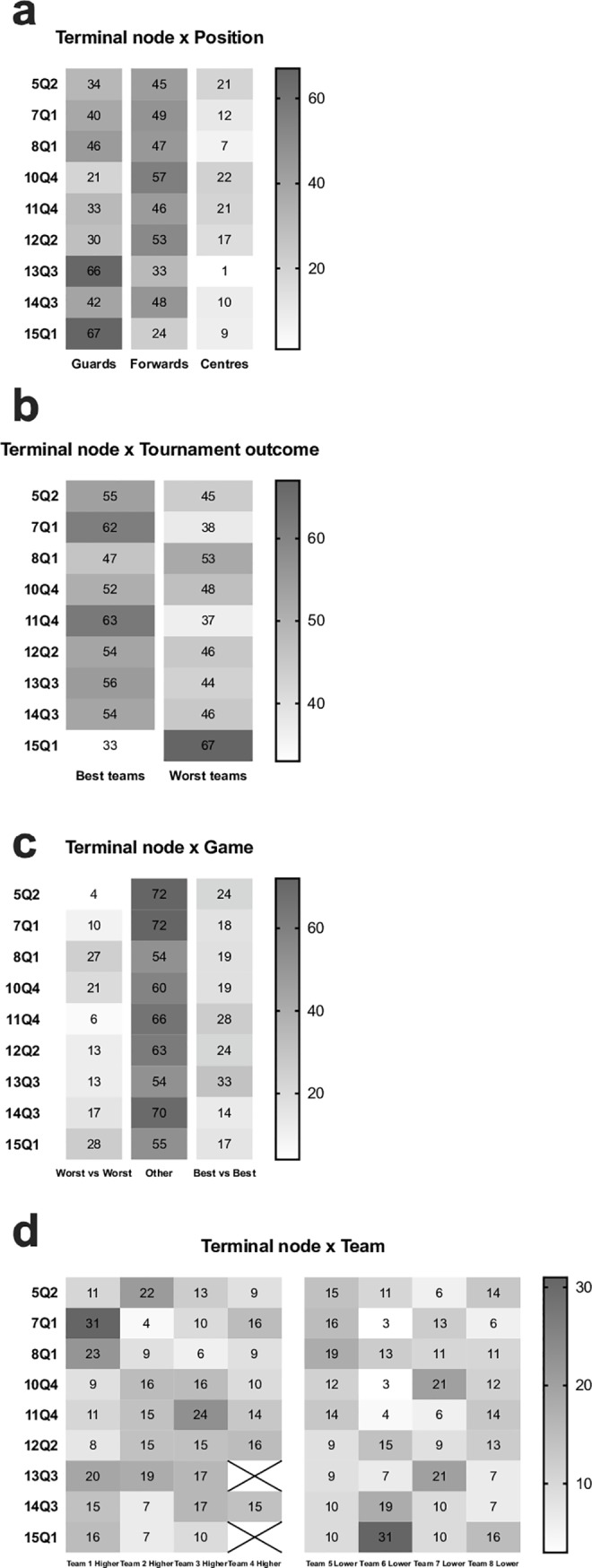
Percentages of distribution from all cases in the terminal nodes, according to playing position (panel a), tournament outcome (panel b), game (c) and team (panel d).

The results from the classification tree were contrasted with playing positions (χ^2^ = 97.1 p<0.0001, Fig 2A), showing that guards were particularly active in terminal nodes 13 and 15, related to the first and third quarters of the game. On the contrary, the centers were more active in terminal nodes 5, 10 and 11, related to the second and fourth quarters of the game. Fig 2B presents the contrast with tournament outcome (χ^2^ = 25.4 p = 0.0013) and shows that worst teams were particularly active in terminal node 15, related to the first quarter performance. Results describing the game type (χ^2^ = 58.4 p<0.0001, Fig 2C) show that best vs best games were more related to performing in terminal nodes that emphasize the third and fourth quarters of the game, whereas worst vs worst games seem more related to terminal nodes that emphasize the first quarter of the game. Finally, it is possible to identify different team profiles (χ^2^ = 195.4 p<0.0001, Fig 2D). For example, team 1 was particular present in terminal node 7 related to performance in the first quarter, whereas team 4 was not present in terminal nodes 13 and 15.

**Fig 3:**
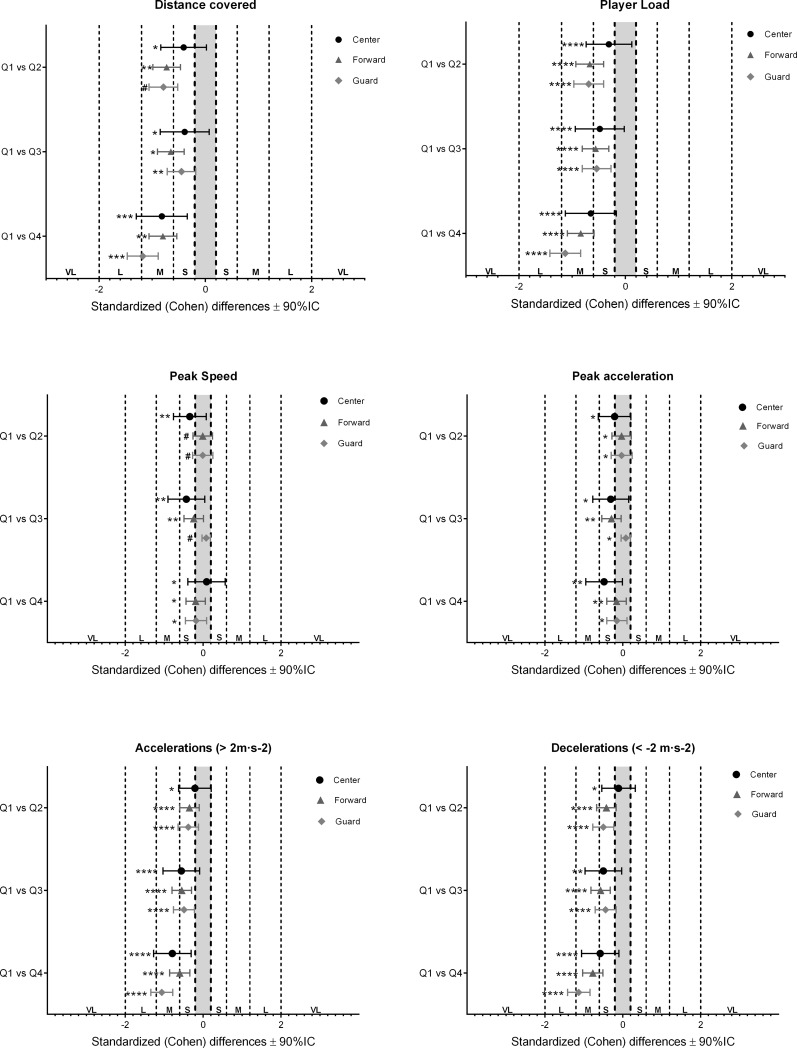
Standardized differences (Cohen’s d) in variables according to the 3 positions and game quarter. **Error bars indicate uncertainty in the true mean changes with 90% CI.** #: very unlikely; *: unlikely; **: possible; ***: likely; ****: very likely; *****: almost certainly. VL: Very Large; L: Large; M: Moderate; S: Small.

Magnitude-based inferences analysis are shown in Fig 3. Distance covered, Player Load, accelerations (> 2 m·s^-2^) and decelerations (< -2 m·s^-2^) show moderate changes and *very likely-almost certain* decreases in the last quarter (Q4), whereas peak speed and peak accelerations only showed small changes and *possibly* decreases between quarters for all positions.

## Discussion

Game activity profiles for elite basketball players have previously been reported in a few studies using video analysis systems. To the best of our knowledge, this is the first study to provide detailed information on the physical demands of elite U18 basketball players of different playing positions and to describe the activity during each game quarter. The main finding of the current research suggests an overall decrease in physical demands such as distance covered, player load, number of high intensity accelerations and decelerations between the first and last quarter of the games for all playing positions.

The classification three was able to model 41% of the results, predicting better the physical performance from the first and the fourth game quarters, using the total distance covered as the most accurate variable. Results obtained seem to suggest that entering the game in the first quarter is more physically demanding because the teams are still co-adapting to the competition environment and this search and attunement will likely result in good and bad decisions and movement patterns. Available research suggests that expert players (basketball all-star players) may well make less mistakes when deciding when and where to run, possibly taking better and shorter paths to reach their destinations and technical-tactical goals [28]. These fewer mistakes in a game might well result in lower distances covered by these players either in offense and defense [28]. Therefore, this might well help explaining the results obtained in the current study. In addition, it is also possible that in the fourth quarter, the proximity of the final decision of the game increases pressure for players, that results in reducing risks and game pace [28], thus, decreasing the activity demands.

When the classification three was contrasted against situational descriptors, there were interesting trends showing that guards are particularly active in the first quarter, probably because that playmaker role, increases responsibility in the environmental co-adaptation process. Conversely, the centers seem more active during the fourth quarter, where demands for less risk and pace benefits their performance profile, especially taking complex offensive actions near the basketball board [29]. For example, in the fourth quarter stressful moments, it seems more adequate to use set plays that aim to create space-time conditions near to the basket and explore the possibility of shooting the ball in closer positions to the basket. Following the analysis of the situational descriptors, it can be also suggested that worst classified teams were more active during the first period and that higher quality games had predominant activity during the third and fourth quarters. These results seem to fit well the nature of the game and provide the novelty of having concrete threshold scores in each important variable. For example, performance in the first quarter can be described with distance covered higher than 77.5 m·min^-1^, distance covered at low speed (< 6 km·h^-1^) higher that 31.0 m·min^-1^ and high intensity accelerations higher that 2.0. At the end, results also identify each team fingerprint, that result in the interactive effects of all activity and situational descriptors from the game. In fact, each team has different players and circumstances to attend and, the knowledge of all this integrated information can provide the coaching staffs for complementary perspectives on players’ performance.

Previous research showed somehow contradictory results when comparing game quarter demands. In fact, there are studies suggesting that fatigue did not affected the intensity of efforts [30], probably a consequence from the unlimited player substitutions. Conversely, other studies might be suggesting that distance covered at high-intensity activity declines from the first to the last game quarter (843 ± 60 m to 705 ± 21 m, respectively), while distance covered at low intensity activity was kept at very similar values (657 ± 6 m and 657 ± 9 m, respectively) [31]. Furthermore, the live time spent at high-intensity activity has shown significant decreases during the second and last quarter [6], whereas striding and sprinting were reduced during the second half [2]. All these fluctuations can be explained by a combination of physiological (e.g., muscle glycogen depletion, dehydration), tactical (e.g., ball control, game pace), and game-related factors (e.g., scoreline, substitutions, time-outs, player fouls) [31].

The current study showed that the total running performance per minute decreases in all playing position with moderate changes along the first and the last quarter. This consents with reduction in vigorous activities in the fourth quarter reported in a recent systematic review analyzing the activity demands and physiological responses during basketball match-play [32]. Although fatigue could justify in part this reduction, the fact that total distance per minute showed *possible* moderate decreased between first and second quarter it might be time to consider other reasons such as technical and tactical such as slower transitions. Total distance per minute covered between second and third quarter showed a minimal increased and between third and fourth quarter showed very likely moderate decreased.

The number of accelerations (> 2 m·s^-2^) and decelerations (< 2 m·s^-2^) per minute are related with high intensity neuromuscular efforts and showed a *very likely/almost certainly* moderate/large decrease for all playing positions in the last quarter. These results might suggest less teamwork that facilitates the emergence of these actions in contrast with more individual decisions that probably have a consequence of decreasing the overall team physical demands. For example, tactical actions such as hand on hand and screening could be less frequent and have an impact on accelerations and decelerations. The fact that the fatigue could only explain in part the reduction in distance and the number of accelerations and decelerations at high intensity, is sustained by the small reduction at peak speed and peak acceleration between the first and last quarter.

PlayerLoad is a popular representation of whole-body load provided by accelerometers expressed in arbitrary units. Player Load provides the rate of change in acceleration on the body in 3 different planes [8,22] (mediolateral, anterio-posterior, longitudinal PlayerLoad^™^ correlates with the total distance during sport team games [33] and differentiated between small sided games and competition in Basketball [8,22] (ES 1.17). The current study identified that the player load/min decreased in all playing positions, especially between first and last quarter (moderate changes and *very likely* decreases) very similar to the reduction of total distance covered per minute as showed previously [33]. How ever, when the relationships between player load with the others performance variables were analyzed, there were very large correlations with distance covered in all players. This fact highlights the necessity to complement basketball player load variable with other variables to develop a better understanding, specially able to discriminate changes at high intensity activity.

Data collection was performed under the same conditions throughout the games; however, the sports indoor court temperature was not controlled. Another potential limitation in this study is related to the fact that (i) players on court might have changed playing positions and (ii) individual fitness information of players could not be obtained, as for example to allow calculating individualized speed thresholds.

The obtained results can contribute to enhance the coaches’ understanding of physical performance in game context during a tournament. A significant reduction in physical demands occurs during basketball games of under 18 elite players, especially between first and last quarter for players in all playing positions. The greatest physical demands reached during the first quarter are probably related to the fact that when teams started the games, obviously drawing, the starter players try to maintain high intensity activity in order to make differences on the score likely through faster transitions that produce shorter possessions. Furthermore, the type of tournament could also advantage the higher demands at the beginning of the game due to the great necessity to win the game and be successful in the tournament. More research is necessary to clarify better if the lowest motion demands during the last quarter are a consequence of fatigue or a consequence from the scoreline or other tactical reasons.
